# Supplementary material for: Effect of Morphologically Controlled Hematite Nanoparticles on the Properties of Fly Ash Blended Cement
Source: Nanomaterials (Basel). 2021 Apr 14;11(4):1003. doi: 10.3390/nano11041003 (PMC8070749; doi:10.3390/nano11041003)
Supplement: Supplementary file 1 [file nanomaterials-11-01003-s001.pdf]

Supplementary Materials

Effect of Morphologically Controlled Hematite Nanoparticles on the Properties of Fly Ash Blended Cement

Pantharee Kongsat [1,2], Sakprayut Sinthupinyo [3], Edgar A. O'Rear [4] and Thirawudh Pongprayoon [1,2,*]

[1] Department of Chemical Engineering, Faculty of Engineering, King Mongkut's University of Technology North Bangkok, Bangkok 10800, Thailand; pantharee99@gmail.com
[2] Center of Eco-Materials and Cleaner Technology, King Mongkut's University of Technology North Bangkok, Bangkok 10800, Thailand
[3] Siam Research and Innovation Co., Ltd, Saraburi 18260 Thailand; sakprays@scg.com
[4] School of Chemical, Biological and Materials Engineering and Institute for Applied Surfactant Research, University of Oklahoma, Norman, OK, 73019, USA; eorear@ou.edu
* Correspondence: thirawudh.p@eng.kmutnb.ac.th; Tel.: +66-8-9182-6168

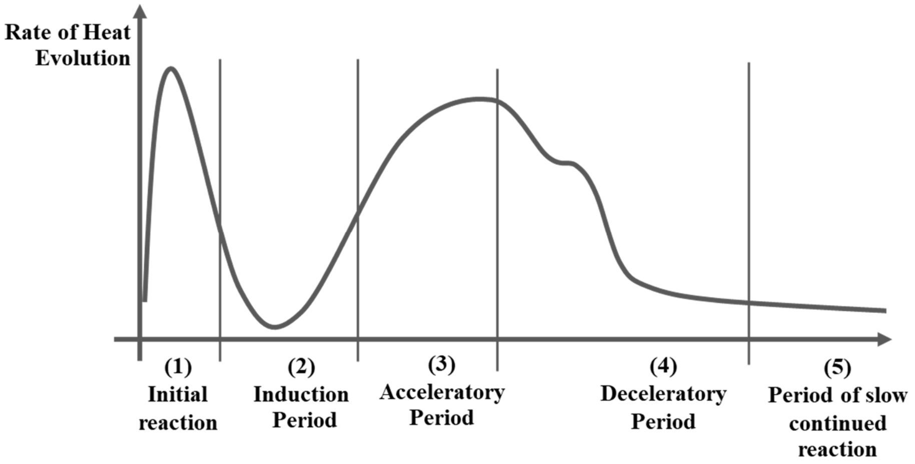

Figure S1. Heat of hydration curve

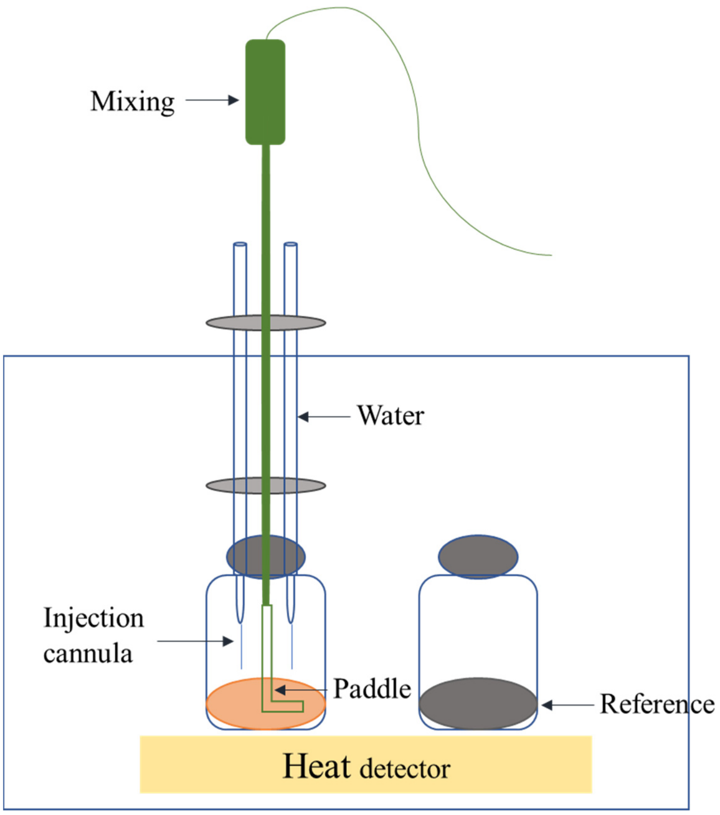

Figure S2. Syringe to fill water to the binder for hydration observation by TAM air calorimeter

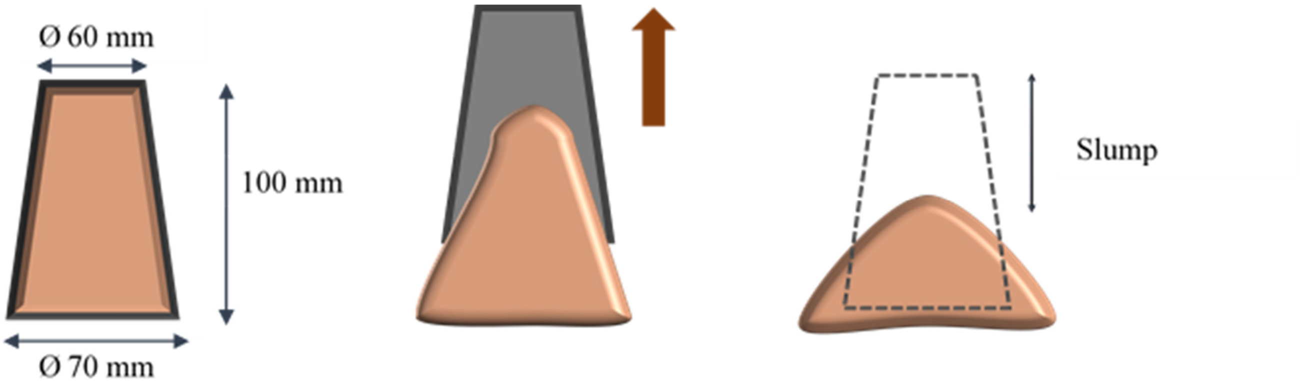

Figure S3. Mini-slump test operation

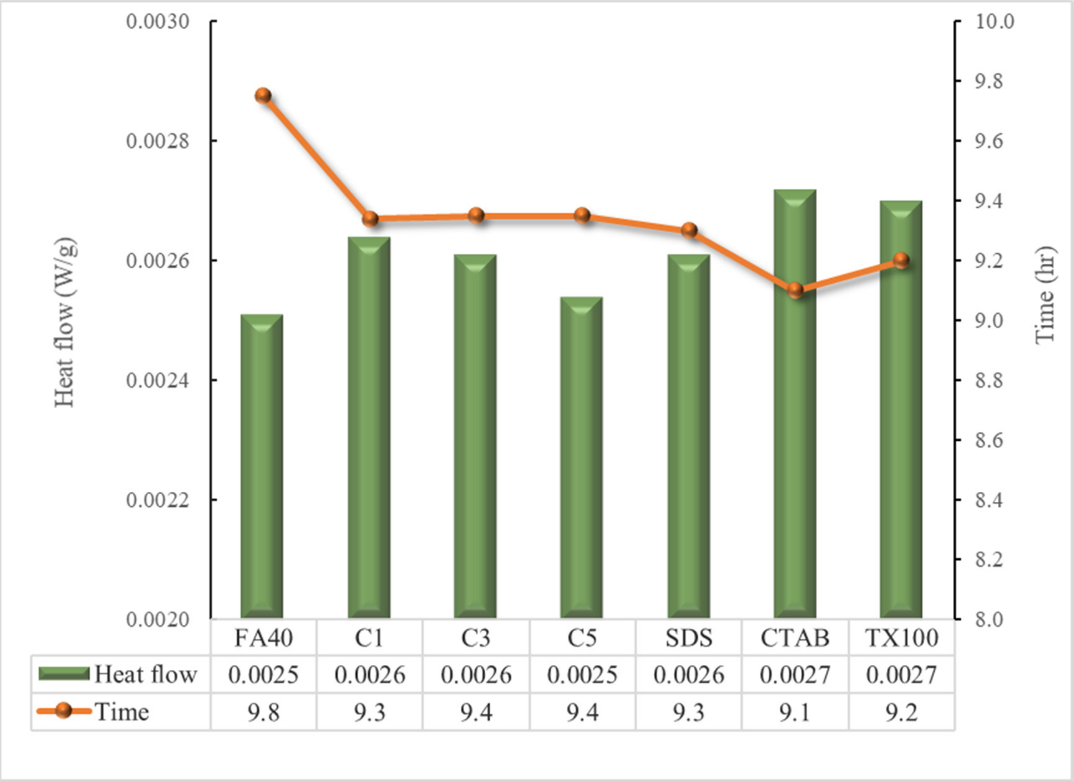

Figure S4 Comparisons the heat flow and time of the hydration peak maximum for FA blended cement with commercial α-Fe_2O_3 and synthesized α-Fe_2O_3
